# Relationship between radiological grading and clinical status in knee osteoarthritis. a multicentric study

**DOI:** 10.1186/1471-2474-13-194

**Published:** 2012-10-09

**Authors:** Daniel Hernández-Vaquero, José Manuel Fernández-Carreira

**Affiliations:** 1 School of Medicine, University of Oviedo, Julian Claveria, 6, 33006, Oviedo, Asturias, Spain; 2 Epidemiology Unit, Hospital San Agustín, Heros s/n, 33400, Avilés, Asturias, Spain

## Abstract

**Background:**

Controversy exists regarding the relationship between radiographic findings and clinical status in knee osteoarthritis. Although the surgical indication for total knee arthroplasty (TKA) should be based on pain, clinical status, and the deterioration of quality of life, the radiographic study is the most commonly used criterion for preoperative evaluation. The objective of this study is to find out the relationship between the Ahlbäck classification and clinical status in patients undergoing TKA.

**Methods:**

1329 protocols were collected from preoperative studies in four multicentric working groups (the Interax, Duracon, Scorpio, and Triathlon Spanish groups) in 30 Spanish hospitals. Mean age was 70.4 years (SD: 6.8; range: 35 to 98); 76.3% of patients were women. Patients entered the study whenever the surgeon found that medical treatment was insufficient to control pain and functional limitation. Data were collected using electronic Case Report Forms, and included Ahlbäck grading scores, Hospital for Special Surgery Knee Score (HSS), SF-12, and other clinical and epidemiologic variables.

**Results:**

According to the Ahlbäck grading system, patients were divided as follows: 243 grade I (18.3%), 358 grade II (26.9%), 416 grade III (31.3%), 241 grade IV (18.1%), and 71 grade V (5.3%). As for HSS, the following scores were obtained: <60 points in 925 patients (69.6%), 60 to 69 points in 286 patients (21.5%), 70 to 84 points in 112 patients (8.4%) and 85 to 100 points in 6 patients (0.5%). Scores showed a statistically significant difference depending on Ahlbäck grade, with a clear tendency towards decrease in HSS scores as the Ahlbäck grade increases (p<0.001). However, the HSS score difference between Ahlbäck grades I and V was of 9.56 points only. Comparing the status of the patients at the start (1994) and at the end (2010) of the data collection process, we observed that patients who underwent surgery in the last years were older and showed a lower Ahlbäck grade.

**Conclusions:**

We found a relationship between Ahlbäck grading and the preoperative clinical score. The range of variability of the HSS score between the different Ahlbäck grades is small.

## Background

Knee Osteoarthritis (OA) is a frequent process especially affecting women over age 50. Among United States adults, the prevalence of knee OA and symptomatic knee OA is 37.4% and 12.1%, respectively (4.3 million older US adults). Although it may affect all kinds of people, it is more frequent in individuals with great body mass index (BMI ≥ 30), greater age, races/ethnicities such as non-Hispanic Blacks, and among men with manual labor occupations [1]. Controversy exists regarding the relationship between radiographic and clinical status in knee OA [2]. Standard radiographs of the knee are an integral part of the primary assessment for osteoarthritis. Although the Ahlbäck classification [3] of knee osteoarthritis is over 50 years old, it is still commonly used in rheumatology and orthopedics for the follow-up of disease progression, and as a classification criterion in clinical trials and epidemiological studies. The Ahlbäck classification is probably the most quoted classification in the literature on preoperative studies and is also used for surgical indications such as total knee arthroplasty (TKA) or unicompartmental knee arthroplasty, arthroscopic debridement, tibial osteotomy, etc. The Ahlbäck system is used often because it is rather simple and rough, and thus a good reproducibility can be expected even when observers are not particularly skilled with its use [4].

TKA is a frequently used procedure for knee arthritis when conservative measures do not work. Although the surgical indication for TKA should depend on clinical status, a radiographic evaluation —following the Ahlbäck criteria— is the most commonly used grading system for preoperative assessment. Some publications by scientific societies [5] have acknowledged the Ahlbäck classification as a valid aid for the selection of the surgical procedure. The Hospital for Special Surgery Knee-Rating Score (HSS) [6] is one the most regularly used questionnaires to assess knee status, both preoperatively and after TKA. This classification system assigns a maximum of 100 points, which are subdivided into six categories: pain, function, range of motion, muscular strength, flexion contracture, and stability. Among the tools for assessing quality of life, the Short Form 12 (SF-12) —more precisely, its mental and physical components— is one of the most widely used, and has been validated in several populations and countries. It is easy to self-complete and it has been employed to follow the changes in health-related quality of life after a surgical procedure, such as a TKA [7].

An advantage of multicentre studies is that they portray the actual situation of the medical practice, as opposed to other types of studies which may come from high-level, specialized centers. The heterogeneity of the surgeons' level of training, age, working situation, and the fact they work at different centers, enrich the results and may inform the health authorities about the usage of a given procedure.

The aims of this work are:

1. To analyze the relationship between the Ahlbäck grade and the HSS and SF-12 scores in a series of knees undergoing TKA included in a multicentric study over a 17-year period.

2. To discover whether there exists any variation in such relationships depending on the patient's gender and age.

3. To find out if there had been any changes in the Ahlbäck grade and the clinical-functional status of TKA patients over the 17-year period.

The ultimate goal of our study is to determine whether classifying patients according to the Ahlbäck grading scale correlates with the clinical and functional status of the patients.

## Methods

During a 17-year period (1994–2010), clinical and radiographic questionnaires of patients with knee arthritis accepted for TKA treatment in four multicenter working groups (Interax, Duracon, Scorpio and Triathlon Spanish groups) in 30 general hospitals were collected. Forty-one surgeons were involved in the study. Different surgeons had different levels of training, and hospitals varied enormously in terms of size, level, and area of influence. Patients entered the study whenever the surgeon found that physical and medical treatments (NSAIDs and analgesics intake for at least six months) were insufficient to control pain and a severe functional limitation was present. Besides at the preoperative assessment, the questionnaire was filled in with data on the intervention and the clinical and radiographic status at six months after TKA and then annually. Patients were excluded from the study if they needed revision surgery or if they did not attend the scheduled appointments. All patients signed an informed consent form which described the suggested surgical procedure, the foreseeable risks, any potential complications, and the possibility of abandoning the study voluntarily. Preoperative and follow-up data were encrypted with a study-specific numeric set. The study was approved by the San Agustín Hospital Research Committee.

**Figure 1:**
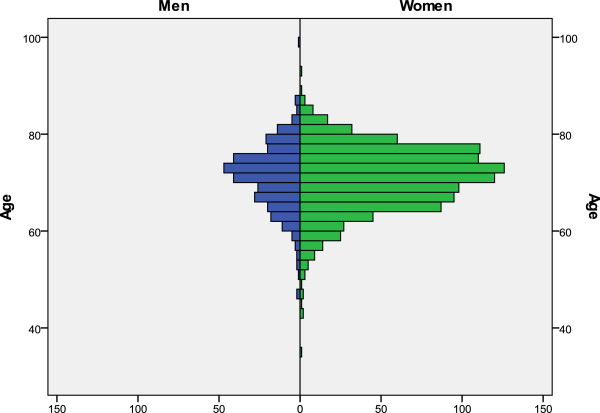
Age distribution by gender.

**Figure 2:**
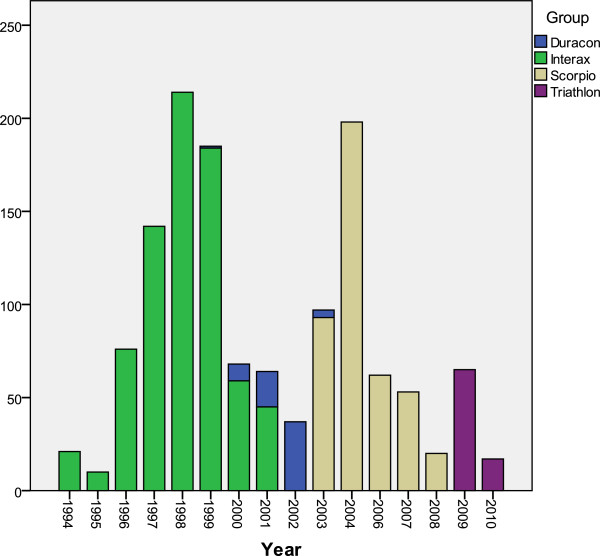
Year of inclusion by group.

Data collection started when the patient was included in the surgery waiting list; at that moment, a clinical and radiographic preoperative assessment was carried out. The surgeon filled in the HSS questionnaire, whereas the patient completed the SF-12 form. Any radiological findings in the radiographic study were recorded. Two projections were examined: frontal and lateral, with the knee fully extended in weight-bearing position. The Ahlbäck grade [3] for each case was agreed upon by the surgeon who added the case to the study and the radiologist who performed the radiographic assessment. Patients who could not achieve full extension of the knee with resting limb were discarded for this study. Out of the total 1717 questionnaires obtained, 1329 cases were assessed; they all had gone through the full preoperative clinical and radiographic assessment. In 577 cases, the mental (MCS) and physical (PCS) component scores of the SF-12 form were also obtained. Mean age of the patients was 70.4 years (SD: 6.8, range: 35 to 98) (Figure 1), and 76.3% were women. Table 1 shows the study group in which the patients were included, and Figure 2 shows their distribution throughout the years of the study.

**Table 1:** Groups and number of TKAs

	**N**	**%**
Duracon	70	5.3
Interax	751	56.5
Scorpio	426	32.1
Triathlon	82	6.2
Total	1329	100.0

Data were collected using electronic Case Report Forms (eCRF), and included Ahlbäck grading, HSS, and other clinical and epidemiologic variables. Statistical analyses were performed with the SPSS v19 software. Descriptive statistics was carried out for all variables. The differences in HSS scores between the five Ahlbäck grades were analyzed with analysis of variance (ANOVA) and the Student's *t*-test when Ahlbäck grading was grouped (grades I to IV versus grade V). The Mann–Whitney non-parametric test was used to analyze the Ahlbäck grade, comparing the first 50 with the last 50 cases. The general linear model was used to analyze the relationship between Ahlbäck and HSS, taking gender as an interacting variable, the HSS score as a dependent variable, and Ahlbäck and gender as fixed factors. Statistical significance was achieved when the p value was <0.05.

## Results

**Figure 3:**
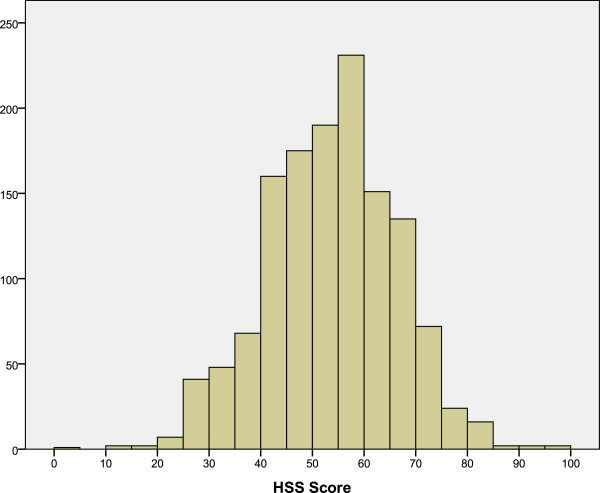
HSS score in overall series.

**Figure 4:**
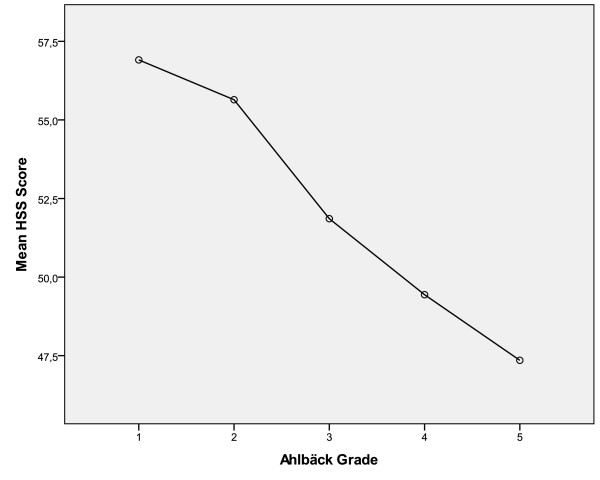
HSS score and Ahlbäck classification relationship.

Ahlbäck grading [3] was distributed into five groups (Table 2). More than 75% of the patients were classified as either grade II, III or IV, and 18.3% were classified as grade I. Regarding HSS, we found an average score of 53.12 points (SD: 12.56): 925 patients showed an HSS score <60 points (69.6%); 286, between 60–69 (21.5%); 112, between 70–84 (8.4%); and 6, between 85–100 (0.5%) (Figure 3). There was a statistically significant difference in the HSS scores depending on the Ahlbäck grade, with a clear tendency towards decrease in HSS scores as the Ahlbäck grade increases (p<0.001, ANOVA) (Table 3). However, the HSS score difference between Ahlbäck grades I and V was of only 9.56 points (Figure 4).

**Table 2:** Ahlbäck grade in the series

**Ahlbäck grading**	**N**	**%**
I Narrowing of the joint space (with or without subchondral sclerosis) (*)	243	18.3
II Obliteration of the joint space	358	26.9
III Bone defect/loss <5 mm	416	31.3
IV Bone defect/loss between 5 and 10 mm	241	18.1
V Bone defect/loss >10 mm, often with subluxation and arthritis of the other compartment	71	5.3
Total	1329	100.0

(*) Joint space narrowing is defined by a space inferior to 3 mm or inferior to half of the space in the other compartment (or in the homologous compartment of the other knee).

**Table 3:** Ahlbäck grade and HSS score

**Ahlbäck grade**	**N**	**Mean HSS**	**SD**	**Standard Error Mean**	**95% confidence interval for mean**		**Min**	**Max**
					**Lower limit**	**Upper limit**		
I	243	56.91	10.750	0.690	55.55	58.27	32	82
II	358	55.64	12.019	0.635	54.39	56.89	22	95
III	416	51.86	12.966	0.636	50.61	53.11	3	95
IV	241	49.44	12.699	0.818	47.83	51.05	18	94
V	71	47.35	12.322	1.462	44.44	50.27	12	73
Total	1329	53.12	12.596	0.346	52.44	53.80	3	95

**Figure 5:**
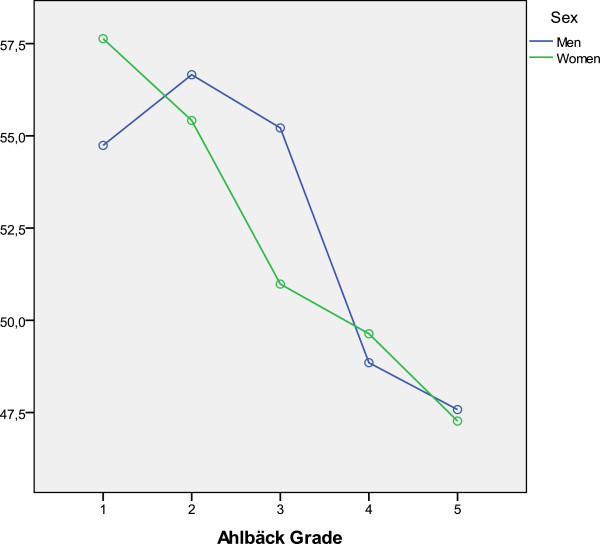
Relation between HSS score and Ahlbäck grade by gender.

No relationship was found between the MCS profile of SF-12 and the Ahlbäck grade, but a significant difference (p=0.006, *t*-test for Equality of Means) was discovered when analyzing the PCS profile differentiating grades I-IV (558 patients) and grade V (19 patients). While studying the relationship between Ahlbäck grade and HSS scores taking into account the gender of the patients, we found that outcomes differed depending on gender (p=0.042), especially in Ahlbäck grade III: men scored 55.2 points and women, 50.9 (mean values) (Figure 5).

**Figure 6:**
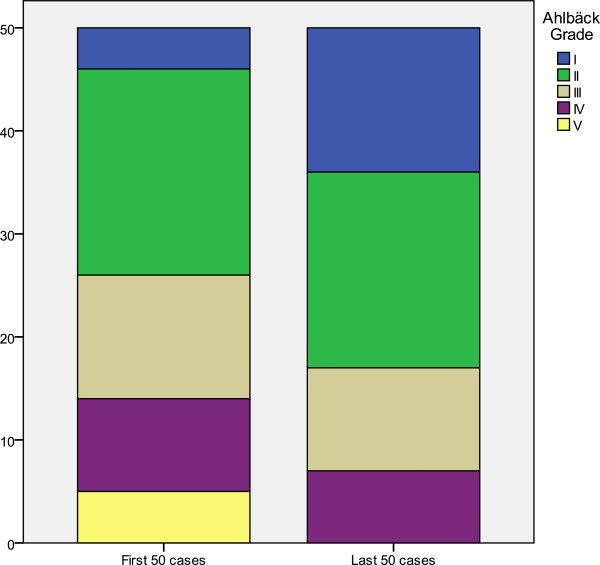
Ahlbäck grade for first and last cases included.

Ahlbäck grade distribution was compared between the first 50 cases (entered into the study between 1994 and 1996) and the last 50 cases (2009 and 2010). As Figure 6 shows, there is a difference in distribution according to the Ahlbäck grade: those patients who entered the study in the later years had knees with lower Ahlbäck grades (P=0.007, U Mann–Whitney). Similarly, there were differences between the mean ages of the patients included at the beginning of the series (68.43; SD: 6.321; range: 52–81) and those entered at the end (70.29; SD: 5.567; range: 59–80) (p=0.038). Analyzing both sets of data allowed us to deduct that patients undergoing TKA surgery in the last years were older and their knees exhibited lesser degrees of radiographic involvement.

## Discussion

It can be stated that the radiographic classification of knee arthritis is based either on the degree of joint space narrowing, as with the Ahlbäck grading [3], or on the presence of osteophytes, such as the classification proposed by Kellgren and Lawrence [8]. Some authors defend the latter [9], arguing that marginal osteophytes are the most sensitive radiographic feature for the detection of osteoarthritis, more than joint space narrowing, subchondral sclerosis, and subchondral cysts. Both classifications show a good correlation [10] and the Ahlbäck system seems easy to apply and suitable for the assessment of medial compartment arthritis of the knee, the most frequently affected compartment in knee arthritis; thus, it appears to be particularly useful for the orthopedic treatment of knee disorders [11].

Although the relationship between radiographic knee OA and the need for TKA is not yet fully established, severe osteoarthritis and range of motion restriction eventually necessitate surgical intervention. It should be mentioned that in our study more than 18% of the patients in the waiting list for TKA had OA of the knee grade I according to the Ahlbäck classification [3], and in grade I cases, the radiographic images were close to normality. On the other hand, if we take into account the HSS score at the start of the study, 112 patients showed a score >70 points, which translates as a mild clinical and functional involvement. Thus, the first finding of our study was the ascertainment that there is a group of patients showing no relevant radiographic findings which are included into the waiting list for TKA implantation. It is possible that other factors such as the social environment, a suboptimal communication between the patient and the physician, the difficulty of assessing pain or the lack of other resolutive therapeutic measures may modify the demand for TKA.

We have observed a good correlation between Ahlbäck grade [3] and the HSS: whenever the former increases, the HSS score decreases. We haven't found in the literature any publications which may support or contradict this concordance. There are several studies that do not find any relationship between knee pain and radiographic changes. Larsson et al. [12] state it is difficult to correlate chronic knee pain, the diagnosis of radiographic OA, and functional capacity. In this article, the proportion of patients with knee pain found to have radiographic OA ranged from 15–76%, whereas in the case of those with radiographic knee OA the proportion with pain ranged from 15–81%. Considerable variation occurred with x-ray view, pain definition, OA grading and demographic factors. Bedson and Croft [13] mention that the results of knee x-rays should not be used in isolation when assessing individual patients with knee pain, and conclude that knee pain is an imprecise marker of radiographic knee osteoarthritis, although the more severe the radiographic osteoarthritis, the more likely there are to be accompanying symptoms.

The HSS scale we used does not assess only pain, but also function and range of motion. Although it cannot be described as a "quality of life scale", it does analyze some activities (such as stair climbing, the need of assistive walking devices, etc.) which are part of daily life. On the other hand, we have observed that there is only a very small difference in HSS score between Ahlbäck grades I and V (only 9.56 points). This would support the idea that the HSS scale does not discriminate radiologic involvement, meaning that a scarce number of radiographic lesions, or small ones, may nevertheless alter the clinical and functional status of the patient. The American Knee Society Score (AKSS) has been validated and is responsive and reproducible. However, it also suffers from high inter- and intraobserver variation when the assessments are performed by less experienced doctors and nurses [14]. Even though we could not get the SF-12 questionnaires from all the patients in our series, we observed that there was no relationship between the grade of radiographic involvement and the mental health component scale scores of the SF-12. Nor did we find such a relationship with the physical component scale, except if we grouped patients with grades I-IV versus patients with grade V according to the Ahlbäck classification. By performing this association, we did find a statistically significant difference. According to these findings, it seems that there is no relationship between radiographic classification and quality of life with the exception of the physical appearance of patients with more advanced radiographic arthritis.

We have not found differences in HSS scores between Ahlbäck grades when taking into consideration the age of patients. As for gender, differences were only present between men and women with grade III. We did not found any explanation for this finding. In the study by Muraki et al. [15] knee pain was strongly associated with joint space narrowing, especially in men, while women tended to experience knee pain even without radiographic OA. The prevalence of knee pain was age-dependent in women, but not in men.

When comparing the status of patients who entered the study in 1994 and in 2010, we observed that nowadays patients are added to the TKA waiting list with lesser Ahlbäck grades and that their age has increased. The reasons behind these changes may have to do with an earlier indication for TKA both on the part of the surgeon and the patient, since it is known this procedure leads to positive results. The greater mean age may be due to the higher survival rate of the patients. In any case, both findings would require a more specific analysis.

Our study has some limitations. Its multicentric nature has the advantage of providing an overview of the actual clinical practice in hospitals and of a heterogeneous group of surgeons, although this may also become a disadvantage, for it could alter the homogeneity of the results and introduce bias in the measurement criteria. Even though all the participating surgeons were familiar with both the Ahlbäck classification [3] and the HSS scale and used them on a regular basis, there is no way of guaranteeing the data have been properly collected. In our series, the radiographic studies were carried out homogeneously with full extension of the knee and resting limb. Even though some degree of flexion is recommended, since this is a multicentric study involving many surgeons, full extension seemed to be easier to reproduce and standardize. Since all radiographs were taken in this position, the overall results should not be compromised nor altered. If the study had been focused on knee OA progression, it would have been indispensable to perform the radiographic study with the knee slightly flexed [16].

Knee OA assessment and grading is far from solved. We did find a relationship between Ahlbäck grades and the preoperative clinical score. Nevertheless, the range of variability of the HSS score between the different Ahlbäck grades is small, a fact that reduces its usefulness in clinical practice. Besides the scales analyzed in this study, other techniques are needed to discriminate the arthritic knee involvement taking into account the commonness of this process and the socioeconomic imbrications therapeutic decisions have on these patients.

## Conclusions

We found a relationship between the Ahlbäck classification scale and the preoperative clinical score, but the range of variability of the HSS score between the different Ahlbäck grades is small. One fifth of the patients who receive a TKA present a grade I in the Ahlbäck scale and over 9.5% exhibit only a mild involvement in the HSS clinical functional scale. Patients added to the surgery waiting list for this type of intervention in the last years show a lesser radiographic involvement and are older than those patients added 17 years ago.

## Competing interests

The authors declare that they have no competing interests.

## Authors’ contributions

DHV conceived of the study, participated in its design and helped to draft the manuscript. JMFC participated in the design of the study, in the data collection process and performed the statistical analysis. Both authors read and approved the final manuscript.

## Authors’ information

Spanish Groups of Knee Arthroplasty Spanish Hospitals that participated in data collection: Axarquía, Vélez-Málaga; Bellvitge, Barcelona; Cabueñes, Gijón; Central Universitario, Oviedo; Centro ICOT, Las Palmas; Clínico, Málaga; Complejo Hospitalario, Albacete; Complejo Hospitalario, León; Costa del Sol, Marbella; Dos de Mayo, Barcelona; Espíritu Santo, Santa Coloma; Francisco de Borja, Gandía; General, Castellón; General, Jerez de la Frontera; Gregorio Marañón, Madrid; Hospital Clínico, Barcelona; Hospital de la Princesa, Madrid; Hospital de Motril, Motril; José Trueta, Gerona; Juan Grande, Jerez de la Frontera; La Paz, Madrid; La Zarzuela, Madrid; Mollet, Mollet; Mutua de Tarrasa, Barcelona; Parc Tauli, Barcelona; San Agustín, Avilés; San Bernabé, Verga; San Camil, Barcelona; Universitario de Tenerife, Tenerife; Virgen de la Cinta, Tortosa.

## Pre-publication history

The pre-publication history for this paper can be accessed here:

http://www.biomedcentral.com/1471-2474/13/194/prepub
